# Enrichment of beneficial cucumber rhizosphere microbes mediated by organic acid secretion

**DOI:** 10.1038/s41438-020-00380-3

**Published:** 2020-10-01

**Authors:** Tao Wen, Jun Yuan, Xiaoming He, Yue Lin, Qiwei Huang, Qirong Shen

**Affiliations:** 1 Jiangsu Provincial Key Lab for Organic Solid Waste Utilization, Key Laboratory of Plant Immunity, Jiangsu Collaborative Innovation Center for Solid Organic Wastes, Educational Ministry Engineering Center of Resource-saving Fertilizers, Nanjing Agricultural University, 210095 Nanjing, China; 2 Vegetable Research Institute, Guangdong Academy of Agricultural Sciences, Guangzhou, 510640 Guangdong, China

**Keywords:** Secondary metabolism, Soil microbiology

## Abstract

Resistant cultivars have played important roles in controlling Fusarium wilt disease, but the roles of rhizosphere interactions among different levels of resistant cultivars are still unknown. Here, two phenotypes of cucumber, one resistant and one with increased susceptibility to *Fusarium oxysporum* f.sp. *cucumerinum* (Foc), were grown in the soil and hydroponically, and then 16S rRNA gene sequencing and nontargeted metabolomics techniques were used to investigate rhizosphere microflora and root exudate profiles. Relatively high microbial community evenness for the Foc-susceptible cultivar was detected, and the relative abundances of *Comamonadaceae* and *Xanthomonadaceae* were higher for the Foc-susceptible cultivar than for the other cultivar. FishTaco analysis revealed that specific functional traits, such as protein synthesis and secretion, bacterial chemotaxis, and small organic acid metabolism pathways, were significantly upregulated in the rhizobacterial community of the Foc-susceptible cultivar. A machine-learning approach in conjunction with FishTaco plus metabolic pathway analysis revealed that four organic acids (citric acid, pyruvate acid, succinic acid, and fumarate) were released at higher abundance by the Foc-susceptible cultivar compared with the resistant cultivar, which may be responsible for the recruitment of *Comamonadaceae*, a potential beneficial microbial group. Further validation demonstrated that *Comamonadaceae* can be “cultured” by these organic acids. Together, compared with the resistant cultivar, the susceptible cucumber tends to assemble beneficial microbes by secreting more organic acids.

## Introduction

Fusarium wilt disease is a persistent and widespread soil-borne disease worldwide. For a long time, breeders have been developing many resistant varieties that generally express high levels of disease resistance genes and/or produce active proteins to defend against the *Fusarium oxysporum* fungal pathogen^1^. In recent years, increasing numbers of studies have shown that certain beneficial microorganisms can be recruited by plants in their rhizosphere to resist the invasion of pathogens^2^. For example, Berendsen et al.^3^ indicated that *Arabidopsis thaliana* could recruit three bacterial species in the rhizosphere upon foliar pathogen infection; Kwak et al.^4^ found that the tomato variety Hawaii 7996 could recruit members of the *flavobacterium* strain TRM1 to suppress disease. Natural disease-suppressive soils even formed via increases in the abundance of beneficial microorganisms (e.g., *Pseudomonas* and *Bacillus*) by monoculture during a long period^5,6^. These beneficial microorganisms can produce secondary metabolites such as 2,4-diacetylphloroglucinol to antagonize pathogens, thus improving the disease-suppressive ability of the soil. Other studies have shown that root exudates such as citric acid, malic acid and fumaric acid could recruit beneficial rhizosphere microorganisms^7–10^. Moreover, it was recently shown that specialized triterpenes from *A. thaliana* could recruit and maintain an *A. thaliana*-specific microbiota^11^.

Plant breeders have begun to consider the contribution of rhizosphere microbes in the development of resistant varieties^9^, including the regulation of the output of root exudates by controlling ABC transporters^12^ and attempt to transfer microorganisms to the next generation by implanting microbes into flowers^13^. Since the interactions occurring in the rhizosphere between plants and microorganisms are relatively complex and variable, breeding work that encompasses the rhizosphere microbiome is progressing slowly.

Due to the different root secretion patterns even within the same crop, the composition of the rhizosphere microbial community is variable. Interestingly, crops sensitive to pathogens tend to form disease-suppressive soils more easily than do resistant varieties^5^. Further studies indicated that traditional susceptible cultivars tend to maintain stronger interactions between plant and beneficial soil microorganisms compared to those of modern resistant varieties^14–17^. Karasov et al.^17^ found that the diversity of *Pseudomonas* in wild *A. thaliana* leaves was abundant. Recently, a study of domesticated plants seemed to reveal a relatively limited microbiota assembly compared to that of their wild counterparts. Furthermore, the genetic diversity of crop microbiota is likely reduced compared to that of wild plants^18^. Plant domestication probably alters root exudate profiles and thus impacts rhizosphere microbial community structure and function^19^. Whether rhizosphere microbial resistance can compensate for the weakness of plant resistance is relatively unknown. Moreover, which kinds of rhizospheric interactions occur during rhizosphere microbial resistance are unclear.

Here, we grew two types of cucumber with contrasting phenotypes (resistant and highly susceptible to Fusarium wilt disease) in the soil and hydroponically to provide controlled and in situ experimental data on these cultivars’ exudates and their rhizobacterial communities. To identify the specific roles of exudate compounds in the recruitment of beneficial bacteria, we employed statistical analyses to evaluate amplicon sequences of the 16S rRNA genes and used nontargeted metabolomics. We aimed to address (i) whether disease-susceptible cultivars have advantages over resistant cultivars in terms of beneficial bacterial enrichment; (ii) whether these interactions are caused by changes in root exudates; and (iii) if so, which types of exudate compounds are responsible for recruitment.

## Results

### Recruitment of rhizosphere bacterial communities of two cucumber cultivars

**Fig. 1:**
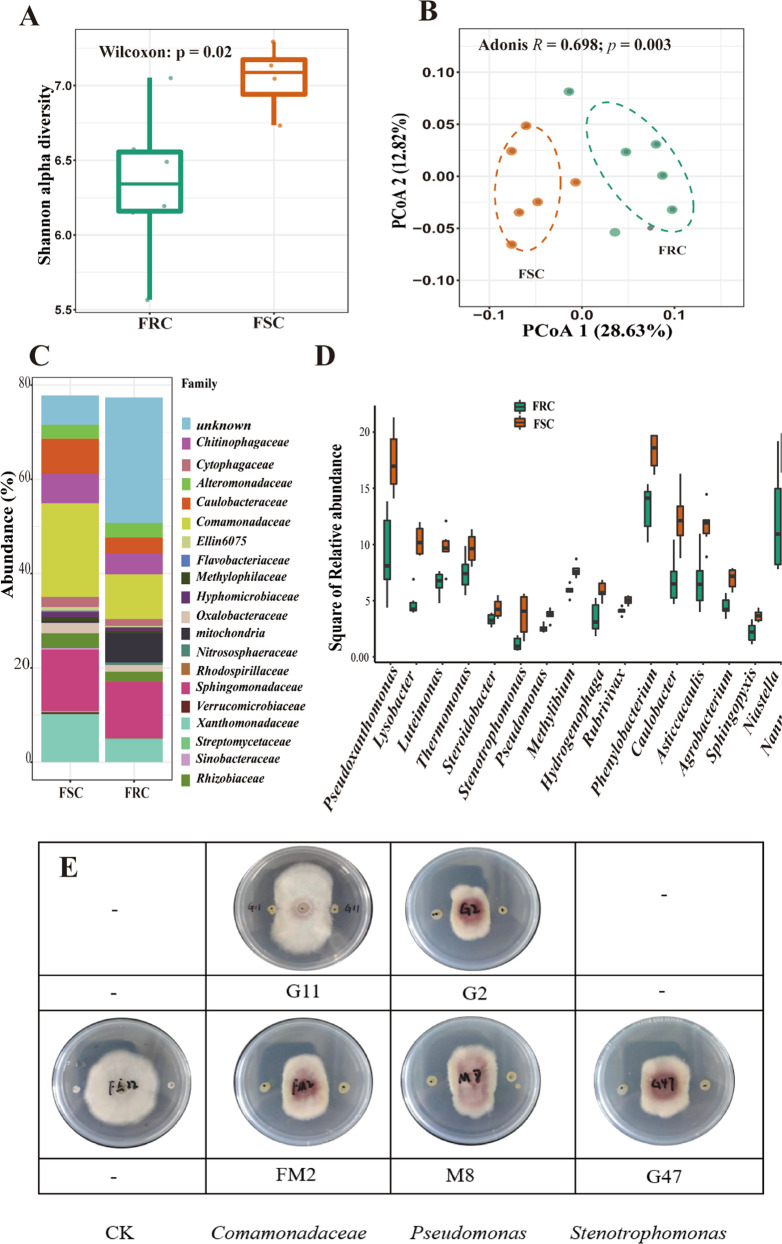
Analysis of rhizosphere bacterial communities between two cultivars. **a** The Shannon-Wiener index of Foc-resistant cultivar (green) and Foc-susceptible cultivar (orange) rhizosphere bacterial communities calculated with all clustered OTUs. The horizontal bars within the boxes represent the medians. The tops and bottoms of the boxes represent the 75th and 25th quartiles, respectively. All outliers were plotted as individual points. **b** Principal coordinate analysis (PCoA) with Bray-Curtis dissimilarity performed on the taxonomic profile (OTU level for a 16S rRNA dataset) of the rhizosphere of the two cucumber cultivars. The *R*-value and *P*-value were evaluated via the Adonis test. **c** Relative abundance (%) of members of the major bacterial phyla, excluding low-abundance OTUs (mean abundance <0.02%), present in the rhizosphere microbial communities of Foc-resistant cultivar (FRC) or Foc-susceptible cultivar (FSC) samples. **d** The relative abundance (%) of members of specific genera enriched in the rhizosphere soil of the FSC and of the entire genus present here was significantly different (*t*-test: *p*<0.05) between the two cultivars. **e** Inhibitory effects of six isolates on *F. oxysporum* belonging to *Comamonadaceae* (G11, FM2), *Pseudomonas* (M8, G2), and Stenotrophomonas (G47).

Bacterial communities in the rhizosphere of two cucumber cultivars were characterized with Illumina HiSeq sequencing. In total, 1,010,289 high-quality sequences were obtained, and each sample contained between 66,236 and 107,307 (84,191 ± 12,531) reads. All the sequences clustered into 8023 operational taxonomic units (OTUs) with 97% similarity. The OTU numbers for each cultivar were 6224 in the Foc-susceptible cucumber (FSC) and 6830 in the Foc-resistant cucumber (FRC), and the most abundant phyla were *Proteobacteria* (64.4%), *Cyanobacteria* (12.2%), *Bacteroidetes* (9.2%), *Acidobacteria* (4.2%), *Actinobacteria* (3.5%), and *Verrucomicrobia* (1.9%). We rarefied (without replacement) 89,948 sequences for each sample to calculate the Shannon index, which is often used to assess the evenness and abundance of microbial communities. The rhizosphere soil of Foc-susceptible cucumber exhibited higher bacterial community evenness than did the Foc-resistant cucumber (Wilcoxon test, *p* < 0.05; Fig. 1a). Principal coordinate analysis (PCoA) with the Bray-Curtis distance showed a significant (*p* = 0.003, *R* = 0.89 in Adonis) difference in the rhizosphere communities between the Foc-resistant cucumber and the Foc-susceptible cucumber (Fig. 1b).

Comparative analysis of the rhizosphere microbiome between the Foc-resistant and Foc-susceptible cucumber cultivars revealed a distinctly different abundance of specific groups at the family level (Fig. 1c), with a higher abundance of the families *Comamonadaceae* and *Xanthomonadaceae* in the rhizosphere of the Foc-susceptible cultivar compared with the Foc-resistant cultivar (Fig. 1c and Supplementary Table 1). At the lower taxonomical level, we found that the abundance of genera belonging to *Comamonadaceae*, likely *Methylibium*, *Hydrogenophaga*, and *Rubrivivax*, and some genera belonging to *Xanthomonadaceae*, such as *Pseudoxanthomonas*, *Lysobacter*, *Stenotrophomonas*, and *Pseudomonas*, were significantly increased in the rhizosphere soil of the Foc-susceptible cucumber compared to the Foc-resistant cucumber (Supplementary Table 2).

We obtained 16 different isolates from the treatments of the FSC and FRC; these isolates belong to the *Bacillaceae*, *Pseudomonadaceae*, *Comamonadaceae*, *Xanthomonadaceae*, *Enterobacteriaceae*, *Oxalobacteraceae*, and *Weeksellaceae*. Through an inhibition assay of *F. oxysporum*, we found that five strains of *Comamonadaceae* (strains G11 and FM2), *Pseudomonas* (strains M8 and G2), and *Stenotrophomonas* (strain G47) could reduce the growth of *F. oxysporum* in vitro (Fig. 1e). It was also found that these five strains obtained more single colonies in the FSC (Supplementary Table 3). Correspondingly, the abundance of genera belonging to *Comamonadaceae* and of the *Stenotrophomonas* and *Pseudomonas* genera significantly increased in the rhizosphere soil of Foc-susceptible cucumber. This suggested that Foc-susceptible cucumber cultivars could recruit more beneficial microbes to resist *F. oxysporum*.

### Root exudate profiles of the two cultivars

**Fig. 2:**
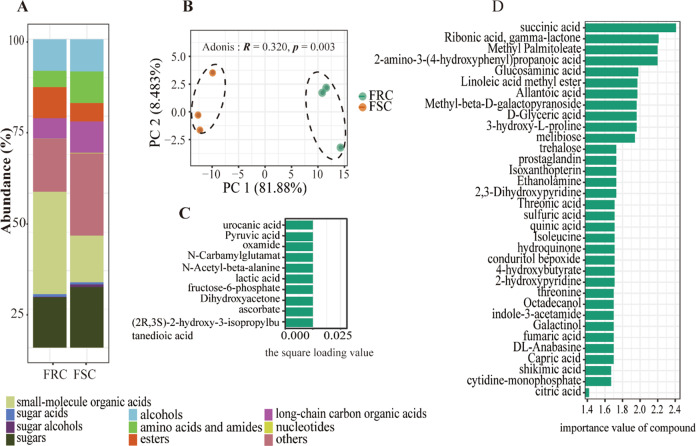
Analysis of exudation profiles between two cultivars. **a** Relative abundance (%) of 10 compounds present in the Foc-resistant cultivar (FRC) or Foc-susceptible cultivar (FSC) root exudates. The data were obtained with peak areas from the GC-MS analysis. **b** PCA plot of the root exudate profiles of two cucumber cultivars evaluated with GC-MS: FRC=rhizosphere of the resistant cultivar; FSC=rhizosphere of the susceptible cultivar. The *P*-values were evaluated via the Adonis test (Adonis: *p*=0.003). **c** The top 10 (top 4% of 236 total compounds) compounds sorted by loading variable importance from the PCA of the two cucumber cultivars. The 10 compounds are ranked in descending order of importance to the loading matrix. **d** The top 35 marker compounds were identified by applying random forest classification of their relative abundances in root exudates of two cucumber cultivars. The marker compounds are ranked in descending order of importance with respect to the accuracy of the model.

Root exudates of the Foc-resistant and Foc-susceptible cucumber cultivars were analyzed by gas chromatography-time of flight mass spectrometry (GC-TOF-MS) after collection from a sterile hydroponic system. A total of 708 chromatographic peaks were detected, and 236 compounds were identified across all the samples. Then, we divided compounds into several categories based on their chemical nature, namely, sugars (28 compounds), sugar alcohols (3), sugar acids (7), small-molecule organic acids (22), nucleotides (4), long-chain organic acids (14), esters (18), alcohols (16), amino acids and amides (28), and others (97; Fig. 2a). The overall exudation patterns of the Foc-susceptible cucumber plants were found to be distinct (*p* = 0.043, *R* = 0.320 in Adonis) from those of the Foc-resistant cultivar (Fig. 2b). The relative abundance of 157 compounds of the 236 total identified compounds was significantly (*p* < 0.05) different between the two cultivars. Specifically, 79 compounds showed higher relative abundance in the FSC, and 78 compounds showed higher relative abundance in the FRC.

To further explore the major compounds that caused the difference in root exudate profiles between the two cultivars, a Principal Component Analysis (PCA) loading matrix extracted and random forests classification approach were applied. Loading matrix of the PC1 axis which explained 81.9% of variance, from principal components analysis (PCA) were extracted to identify the top 10 compounds (top 4% of total 236 compounds) that were important to differentiate the exudate profiles of the two cultivars (Fig. 2c). These compounds included pyruvic acid and lactic acid (short-chain carbon organic acids), two amino acids (*N*-carbamylglutamate and *N*-acetyl-beta-alanine), and six other compounds, including (2 R,3 S)-2-hydroxy-3-isopropylbutanedioic acid, fructose-6-phosphate, urocanic acid, oxamide, ascorbate, and dihydroxyacetone (Supplementary Table 4). For the random forest classifier (R package *RandomForest*, ntree = 1000), the first 35 of the 236 compounds (top 15%) sorted by loading variable importance were selected (Fig. 2d and Supplementary Table 5). Then, the top 10 important compounds selected from the PCA and the top 35 important compounds from the random forest classification were further evaluated for significant differences between the cultivars using a *t*-test with *p* < 0.05 and a log_2_(fold change) <1.5. Finally, 34 compounds were recognized as playing the most important role in the separation of the two root exudate profiles (Supplementary Table 6).

### Functional features of root exudates and functional shifts mediated by microbial alterations in the rhizosphere of FSC

**Fig. 3:**
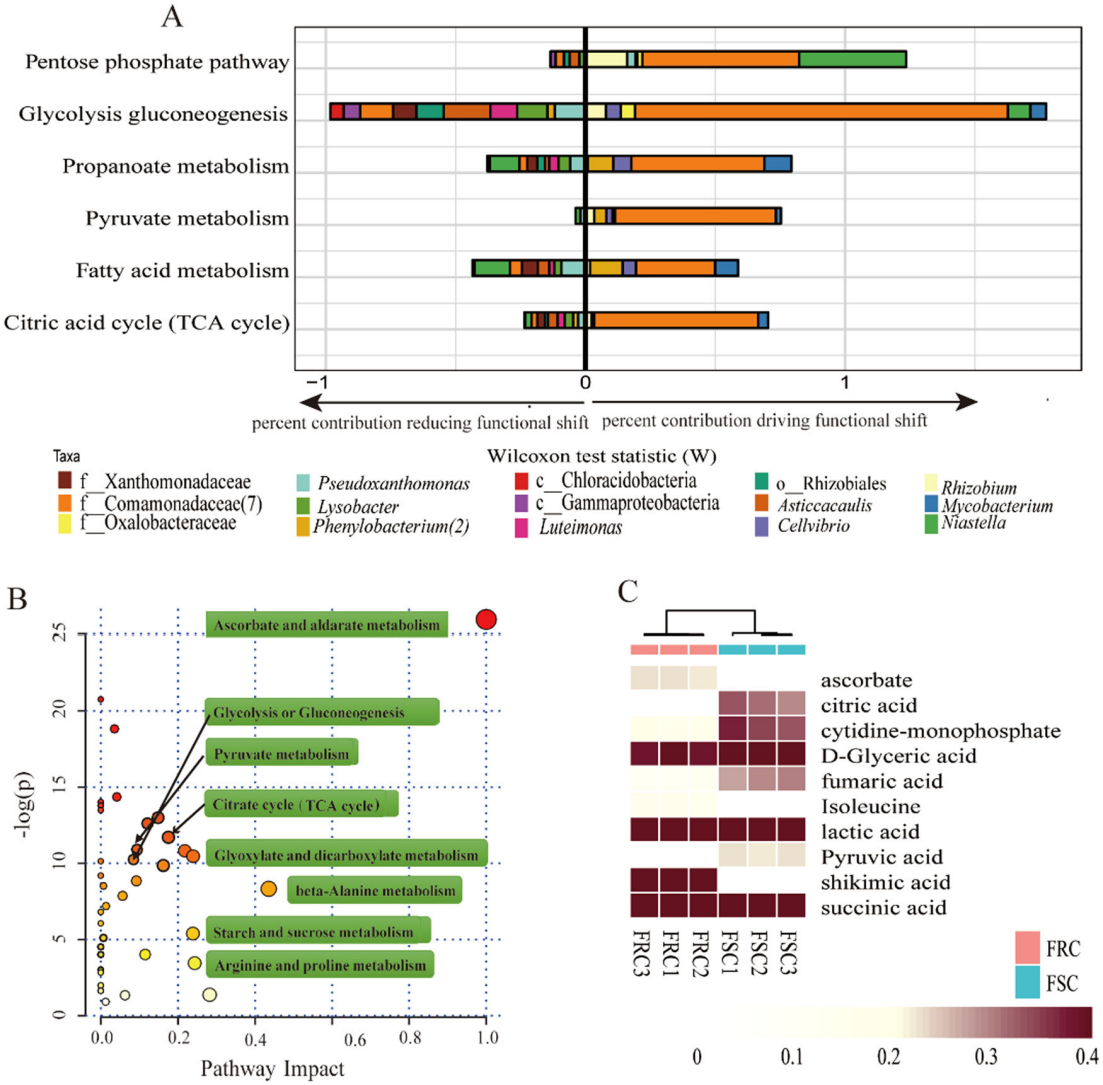
Prediction of the major pathways mediated by microbial communities and KEGG pathway enrichment analysis of root exudates between two cultivars. **a** To identify the shifts in rhizosphere communities caused by these potential beneficial bacteria enriched in the Foc-susceptible cultivar rhizosphere, deconvolution of significant community-wide functional shifts into individual taxonomic contributions was performed. The right bar plot represents relative contributions driving functional shifts by the taxa of Foc-susceptible samples, and the left bar plot represents relative contributions reducing functional shifts by the taxa of Foc-susceptible samples. **b** Different metabolic pathways of the root exudates of two cucumber cultivars. Each point represents a different metabolic pathway, and the size of each point represents the degree of change in each metabolic pathway. **c** Heatmap analysis of 10 compounds selected by PCA, random forest classification, and pathway enrichment analyses; these compounds were significantly (*t*-test, *p*<0.05) different in terms of their relative abundance between the root exudates of the two cucumber cultivars.

It was shown that root exudates were significantly associated with rhizosphere microorganisms according to Mantel’s test (*R* = 0.5966, *p* = 0.003, Supplementary Table 7). Pathway enrichment analysis was performed to further exploration whether root exudates of the Foc-susceptible cultivar are involved in the recruitment of beneficial bacteria. The results revealed that small organic acid metabolism pathways (such as pyruvate acid metabolism and the citric acid cycle) and amino acid metabolic pathways (valine, leucine and isoleucine biosynthesis and glycine, serine and threonine metabolism) were significantly (Wilcoxon test: *p* < 0.05) different (Fig. 3b and Supplementary Table 8). Then, 24 compounds involved in these different pathways were selected to check if they were covered in the 34 represented compounds causing major differences in the root exudate profiles. Ten compounds were ultimately selected to represent the core difference in root exudate profiles and metabolic pathways (Supplementary Table 9). Among these 10 compounds, six were small organic acids, including four that mainly participate in pyruvate acid metabolism and the citric acid cycle (Fig. 3c).

To determine whether these ten compounds or core differential pathways influenced the assembly of rhizosphere microbial communities and variation between the two cultivars, we employed the FishTaco framework for microbial communities to predict the functional information within the microbial communities and to determine the causes of the shifts in rhizosphere microorganisms. Marked upregulation of both protein synthesis and secretion and bacterial chemotaxis were observed (Wilcoxon test: *p* < 0.05) for the Foc-susceptible cultivar (FSC), which may be related to the rapid response of soil microorganisms to the roots and corresponded to the enrichment of OTUs from members of the *Comamonadaceae* and *Oxalobacteraceae* families (Supplementary Fig. 1). Many amino acids and small-molecule fatty acids were also more abundant in the rhizosphere of the FSC, including the citric acid cycle. The results showed that enrichment of the citric acid cycle was mainly attributed to shifts in the relative abundance of the *Comamonadaceae* family, as this family contains many genes involved in the citric acid cycle (Fig. 3a and Supplementary Fig. 2). Moreover, the pathway enrichment analysis results based on root exudate data showed that the selected small-molecule organic acids of 10 core root exudates were enriched in the root exudates of the Foc-susceptible cucumber (Supplementary Table 6) associated with the citric acid cycle. Therefore, we suspect that the four organic acids may promote recruitment of *Comamonadaceae* in the rhizosphere of the Foc-susceptible cultivar by enriching compounds in the TCA cycle.

### Impacts of selected small-molecule organic acids on *Comamonadaceae* members

**Fig. 4:**
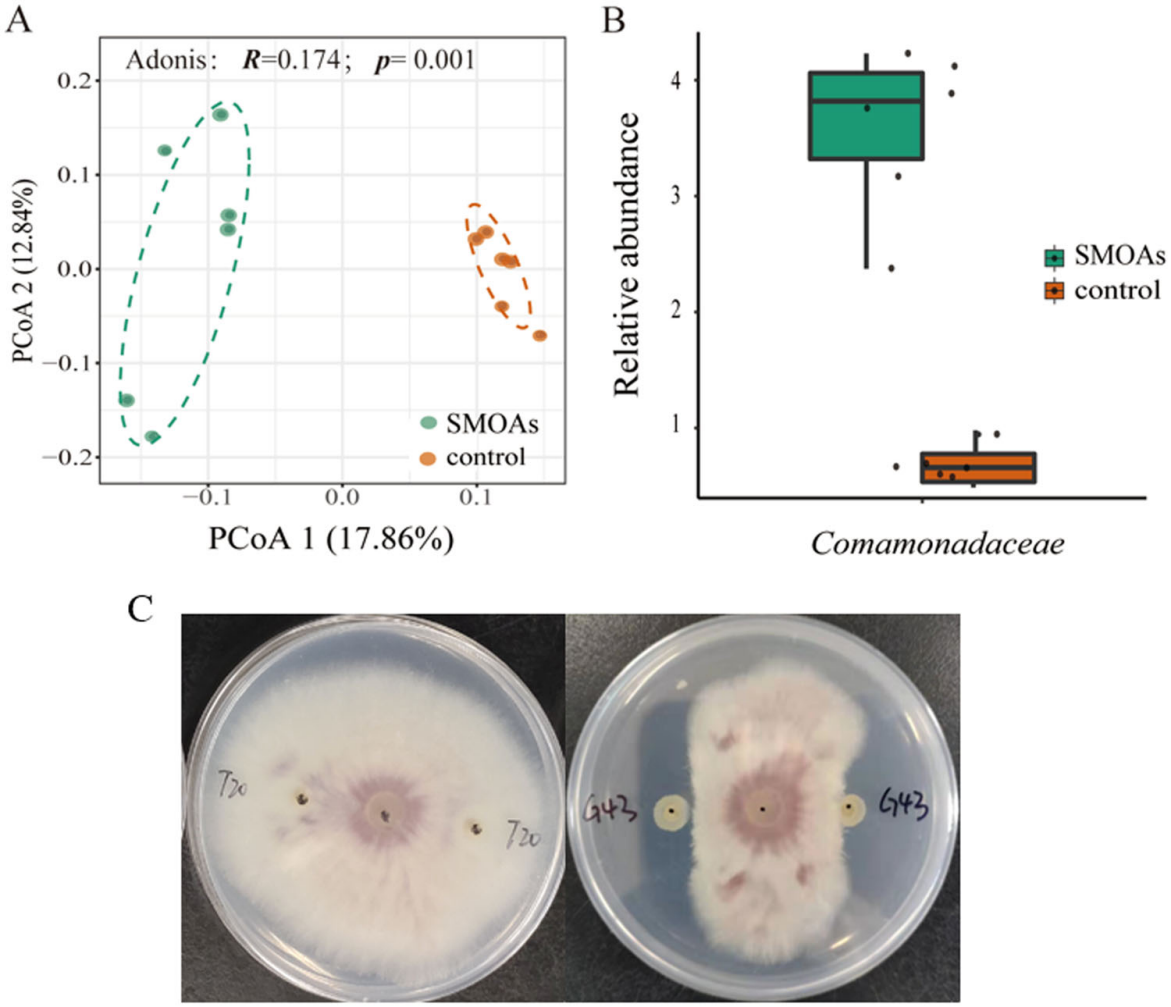
Diversity analysis of bacterial communities in soil treated with different compounds. **a** Principal coordinate analysis (PCoA) with Bray-Curtis dissimilarity of the microbial community in soil treated with four small-molecule organic acids (SMOAs) and the control at the OTU level. The *R*-value and *p*-value were evaluated via the Adonis test. **b** Relative abundance (%) of *Comamonadaceae* enriched in the soil treated with SMOAs (*t*-test: *p* = 0.001). The horizontal bars within boxes represent the medians. The tops and bottoms of the boxes represent the 75th and 25th quartiles, respectively. All the outliers were plotted as individual points. **c** Inhibitory effects of *Comamonadaceae* strain G43 on *F. oxysporum.*

Four small-molecule organic acids that were significantly enriched in the exudates of the Foc-susceptible cultivar were mixed together and repeatedly added to condition the soil. Sequencing results of the communities produced a total of 632,721 sequences, with 15,344–63,539 (35,151 ± 14,705) reads per sample. Higher evenness measures were observed for the rhizosphere soil of the control treatment compared to the SMOA treatment (Fig. 4a, Wilcoxon test: *p* = 0.031). Principal coordinate analysis (PCoA) showed a clear difference (Adonis: *p* = 0.003, *R* = 0.17) in rhizosphere community composition between SMOAs and control (Fig. 4b). Comparative analysis of the microbiome between the SMOAs and control revealed a distinct differential abundances of specific family groups (Supplementary Table 9), including higher relative abundance of the family *Comamonadaceae* in the soil after the application of the four small-molecule organic acids (Fig. 4b).

In total, we obtained 14 different isolates (Supplementary Table 11) from the SMOAs conditioned and control soils, which belong to *Bacillaceae*, *Pseudomonadaceae*, *Comamonadaceae*, *Xanthomonadgaceae*, *Sphingomonadaceae*, *Burkholderiaceae*, *Alcaligenaceae*, *Oxalobacteraceae*, and *Rhizobiaceae*. We then found that *Comamonadaceae* strain G43 had a strong inhibitory effect on the growth of Foc (Fig. 4c). Interestingly, more single colonies were isolated, similar to strain G43, from the SMOA-conditioned soils than from the control soils (Supplementary Table 11). Further alignment indicated that strain G43 was the same as *Comamonadaceae* strain G11.

## Discussion

The rhizosphere microbiome is the first line of soil pathogen defense and plays a vital role in the prevention of pathogen invasion. Among the reported mechanisms, the evenness and richness of the rhizosphere microbiome are central players in providing stability and resilience to stress and invasion^2,20^. High evenness was observed in the Foc-susceptible rhizosphere, which could be attributed to a relatively high variety of exudates supporting more microbial niches. If these niches are left nonstimulated/uninhabited in the Foc-resistant rhizosphere, this could provide a window for successful invasion by other pathogens^21^. In this study, the higher evenness found in the Foc-susceptible rhizosphere compared with the Foc-resistant rhizosphere may result from sufficient coevolution between the host plant and soil microbiome. In other words, the soil microbial metabolic capacity for the resources from FRC roots has not developed^21^. It has been shown that traditional cultivars tend to maintain relatively high evenness of rhizosphere microbiomes^17,22^ and relatively strong interactions between plants and the environment^21^. For instance, mycorrhizal associations have been shown to be increased in older wheat cultivars compared to modern landraces^14^. Similarly, Germida et al.^23^ found that ancient landraces could recruit a more diverse rhizosphere bacterial community than could modern cultivars. We also found higher abundances of *Comamonadaceae* and *Xanthomonadaceae* in the Foc-susceptible cultivar rhizosphere community than in the Foc-resistant one, both of whose members have been reported to be abundant in disease-suppressive soils^24–26^. *Xanthomonadaceae* genera falling within this family, such as *Pseudoxanthomonas*, *Lysobacter*, and *Stenotrophomonas*, have been used as biocontrol agents of several pathogens, including *Fusarium oxysporum*^27–30^. It has been widely observed that fluorescent *Pseudomonas* species produce the antifungal compound 2,4-diacetylphloroglucinol (DAPG) to resist pathogens^5^. All these beneficial genera were more abundant for the Foc-susceptible cultivar than for the Foc-resistant cultivar, and five strains of *Comamonadaceae* (strains G11 and FM2), *Pseudomonas* (strains M8 and G2), and *Stenotrophomonas* (strain G47) showed strong inhibitory effects on the growth of pathogens in vitro (Fig. 1e), which may contribute to the resistance of other pathogens and natural attenuation of *Fusarium oxysporum*. Previous research has shown that crops sensitive to pathogens tend to form disease-suppressive soils more readily than do resistant varieties when under continuous pathogen attack^5^; this phenomenon was supported by our results, as more beneficial bacterial groups were recruited in the rhizosphere of the susceptible crop than in that of the resistant crop. However, some researchers have reported that, compared with susceptible varieties, resistant varieties can recruit more beneficial microorganisms^31^. Recently, Mendes et al.^32^ investigated the composition of the rhizosphere bacterial community of common bean cultivars with different levels of resistance to the fungal pathogen *F. oxysporum* and found that beneficial bacteria, such as *Bacillaceae*, *Pseudomonadaceae*, *Solibacteraceae*, and *Cytophagaceae*, were abundant in the rhizobacterial community of the FRC cultivar. On the other hand, our study showed that the FSC cultivar was more enriched in beneficial rhizosphere microbes. This is probably due to the different crops and cultivars used and the mechanism by which resistance breeding influences plant physiology. More work needs to be done with other crop species.

Root exudates are important for regulating and controlling the composition and function of rhizosphere microorganisms. Previous research has shown that plant species, even different genotypes of the same species, may vary in terms of their rhizosphere microbiome composition and root exudates^19^. In our experiment, the root exudate profiles varied between the FSC and FRC. Interestingly, four small-molecule organic acids (citric acid, fumaric acid, succinic acid, and pyruvic acid) were observed to be the main driving force for the separation of the two exudate patterns and were produced in higher quantities by the FSC compared with the FRC. These small-molecule organic acids reportedly impacted specific beneficial bacterial groups, such as crop growth-promoting rhizobacteria. For example, malic acid and citric acid could chemotactically attract *Pseudomonas fluorescens* WCS365, which could competently colonize tomato roots. Infection of *Pseudomonas syringae* pv. into *Arabidopsis* could induce root secretion of malic acid and thus promote colonization and biofilm formation on the root surface by strain FB17^7,33^. Similarly, watermelon roots could secrete citric acid and malic acid to induce root colonization of the plant growth-promoting rhizobacterial strain *Paenibacillus polymyxa* SQR-21^34^. In our experiment, the four small-molecule organic acids added to the soil enriched the relative abundance of *Comamonadaceae*, an important beneficial bacterial group, and *Comamonadaceae* strain G43, whose sequence is the same as that of G11, showed a strong inhibitory effect on the growth of Foc. These results showed that FSC enriched more beneficial bacteria by regulating root exudates.

Indeed, plants employ complex defense strategies to protect themselves from infection by pathogens. Some physical structures and chemical components of plants show antidisease effects, such as cell wall keratin, wax deposition, lignin, special pores, water holes, and lenticels and induction of various pathogenesis-related (PR) proteins, such as chitinase and glucanase^35^. Additionally, defense responses are induced by plants; these responses mainly include the release of various reactive oxygen species, the expression of defense genes and the development of the hypersensitive response (HR)^36,37^. In recent years, a consensus has gradually been reached in which rhizosphere microorganisms help plants resist pathogens via the secretion of antimicrobial substances, forming biofilms, and via competition for space and nutrients by occupying pathogen niches^38^.

**Fig. 5:**
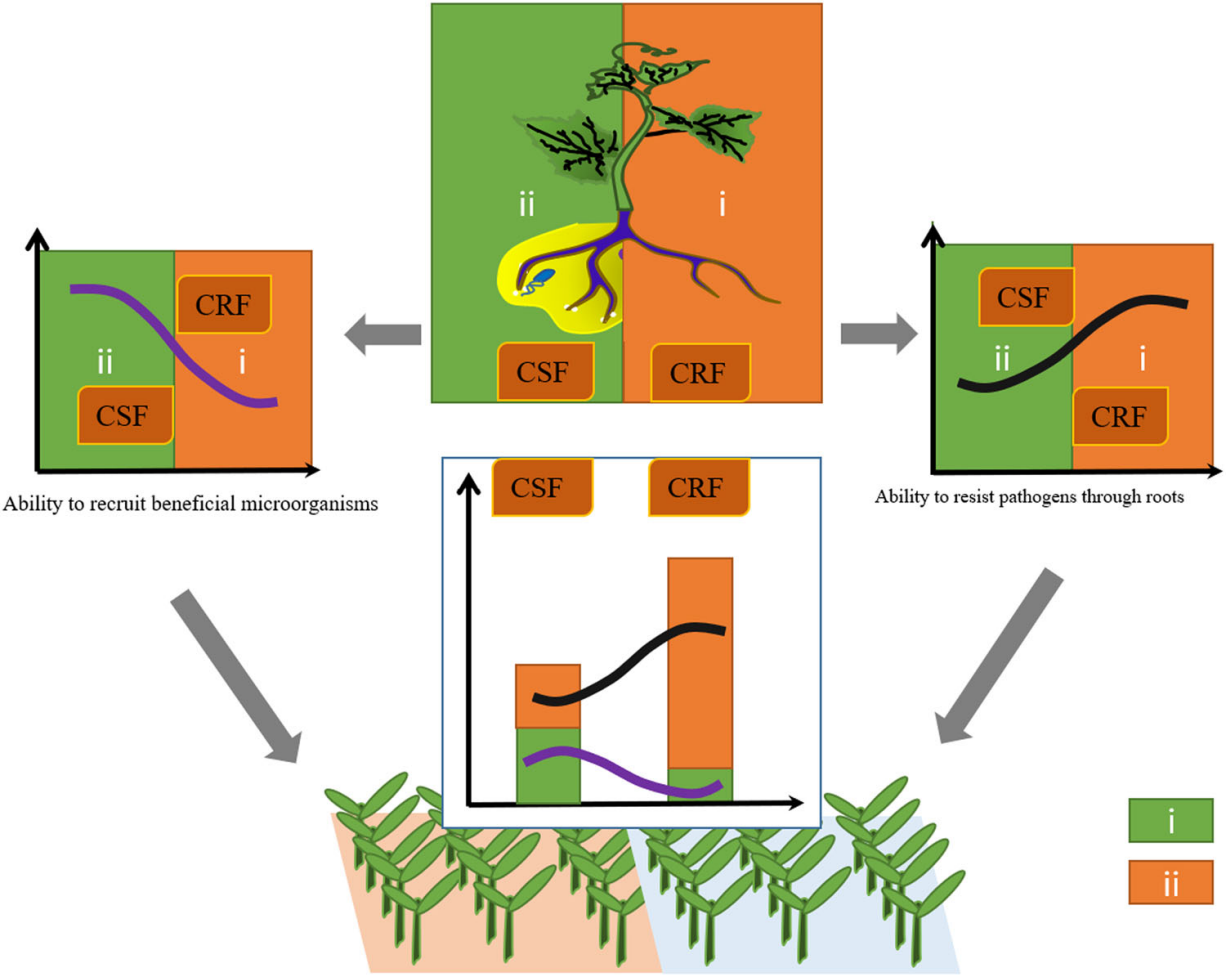
The disease resistance of Fusarium wilt by crops is normally divided into two strategies: strategy i (green) and strategy ii (orange). These strategies are illustrated here, and the resistance level of the two strategies in the Foc-resistant cucumber and Foc-susceptible cucumber (FSC) are shown. In strategy i (plant resistance), plants act as executors by improving physical structures and secreting various new chemical components. In strategy ii (rhizosphere resistance), rhizosphere microorganisms recruited by plants act as executors to confront pathogens by perceiving pathogens, activating the plant immune system, and secreting various effective antimicrobial chemicals.

Direct and indirect (via microbial associations) plant pathogen defense strategies coexist. We divided the concept of plant responses to soil-borne pathogens into two aspects: (i) plant resistance, in which plants act as executors by improving physical structures and secreting various new chemical components; and (ii) rhizosphere resistance, in which rhizosphere microorganisms recruited by plants act as executors to confront pathogens by perceiving them, activating the plant immune system, and secreting various effective antimicrobial chemicals (Fig. 5). In this study, two cucumber cultivars displaying different Fusarium wilt resistance were used to assess the level of these two different strategies. The fundamental data indicated that the FRC showed stronger disease resistance than did the FRC (<15% vs >60% of disease incidence), especially plant resistance (Supplementary Table 12), while stronger rhizosphere resistance was found in the FSC than the FRC. In recent years, breeders have been focusing on the role of microorganisms in plant disease resistance. Owing to the complexity of microbial community-plant interactions and uncertain influences of root exudates on rhizosphere microorganisms, compared with strategy i, strategy ii has received less attention by plant breeders. However, improved tools and reduced costs associated with microbiome analyses can add new standards for plant breeding programs. For example, plant phenotypes that target beneficial rhizosphere microbiomes can be evaluated by profiling root exudates or by using community compositional profiles (i.e., diversity and evenness metrics) as criteria to evaluate a cultivar.

## Conclusion

In this experiment, using high-throughput sequencing, we determined the rhizosphere microbial communities of two cucumber cultivars with different resistance to Fusarium wilt. The evenness of the rhizosphere microbial community of the Foc-susceptible cucumber cultivar was higher than that of the Foc-resistant cultivar; specifically, the relative abundance of *Comamonadaceae* and *Xanthomonadaceae* was greater in the Foc-susceptible cultivar than in the Foc-resistant one. At a lower taxonomic level, higher abundances of the genera *Pseudoxanthomonas*, *Lysobacter*, *Stenotrophomonas*, *Pseudomonas*, *Methylibium*, *Hydrogenophaga*, and *Rubrivivax* were found in the rhizosphere soil of the Foc-susceptible cultivar compared with the resistant cultivar. As an important medium in the rhizosphere microbial community-plant interaction, different root exudation patterns were found between Foc-resistant and Foc-susceptible cucumber cultivars. The small-molecule organic acids pyruvate acid, citric acid, succinic acid, and fumarate in the cucumber root exudates of susceptible cultivars could recruit *Comamonadaceae*, which has been shown to be abundant in disease-suppressive soils in many studies. Taken together, our results indicate that the susceptible cucumber cultivar could enrich beneficial microbes (rhizosphere resistance) to compensate for the weakness of the “plant resistance” to pathogens; this overall process may be caused by regulation of the root exudate profile.

## Materials and Methods

### Soil sampling, plant material, and rhizosphere sampling in the pot experiment

A pot experiment was conducted to evaluate the rhizosphere bacterial communities of two cucumber cultivars with different resistance levels to Fusarium wilt disease. The soil was collected from Baimao town, Changshu city, China (31°35′36.19″N, 120°54′54.93″E, ~300 km away from Nanjing) with no history of cucumber cultivation. The topsoil (20 cm) was air dried, sieved (2-mm sieve) to remove plant debris and rocks, homogenized and stored in plastic bags at room temperature. The seeds of two cucumber cultivars, Foc-resistant cucumber (FRC) Lifeng (disease incidence <15%) and Foc-susceptible cucumber (FSC) B80 (disease incidence >60%), were provided by the Vegetable Research Institute, Guangdong Academy of Agricultural Sciences, China. Before planting, the cucumber seeds were surface sterilized with 75% ethanol for 30 s followed by 5% NaClO for 5 min. The sterilized seeds were placed in Petri dishes with wet autoclaved filter paper in a growth chamber (25 °C, 70% relative humidity in the dark). After 2 days of pregermination, the seedlings were transferred to 200 mL pots (5 cm × 8 cm × 5 cm) filled with 150 g of soil (one seedling per pot). There were 18 seedlings of each cultivar (6 replicates and 3 pots per replication) potted, for a total of 36 pots; the pots were then randomly placed in a growth chamber (28/26 °C day/night cycle, 70% relative humidity, and 180 μmol light m^−2^ s^−1^) and irrigated as needed. The plants were killed at the early flowering stage (30 days after planting), and roots with closely attached soil were harvested from the pot after removing the loosely attached soil by shaking. Three cucumber plants of each replicate were pooled into one sample. In total, 12 rhizosphere soil samples were obtained (2 cucumber cultivars × 6 rhizosphere soil samples) and stored at −70 °C for further microbiota analysis.

### DNA extraction, 16S rRNA gene amplification and amplicon sequencing

Before DNA extraction, 0.5 g of roots with tightly adhered soil was placed into a 2-mL centrifuge tube containing 1 mL of phosphate-buffered saline solution and several sterilized glass beads, after which the mixture was vortexed at maximum speed for 15 min. The suspension (without root materials) was then transferred to a new 2 mL centrifuge tube and centrifuged for 30 min at 15,000 rpm. The supernatant was discarded, and the precipitate was used for DNA extraction. Total DNA was extracted from the precipitate using a Power Lyzer PowerSoil DNA Isolation Kit (Qiagen, Germany) according to the manufacturer’s protocol. The DNA quality and quantity were measured via a 1.2% agarose gel and a NanoDrop 1000 spectrophotometer (Thermo Scientific, USA).

For taxonomical profiling of the bacterial communities, PCR of the 12 DNA samples that targeted the 16S rRNA gene (V4 region) was performed. The primers 515F/806R (F: GTGYCAGCMGCCGCGGTAA; R: GGACTACNVGGGTWTCTAAT) were used for PCR^39^, with an amplicon length of ~292 bp. For amplification, 50 μL reaction mixtures consisted of 1 μL of each primer (10 μM), 25 μL of sterilized ultrapure water, 25 μL of 2× Premix Taq (Takara Biotechnology, Dalian Co., Ltd., China), and 3 μL of DNA (20 ng/μL). A Bio-Rad S1000 (Bio-Rad Laboratory, CA) instrument was used to perform PCR amplification with the following amplification procedure: 95 °C for 5 min; 30 cycles of 94 °C for 30 s, 52 °C for 30 s, and 72 °C for 30 s; and then 72 °C for 10 min. The PCR products were run on a 1.2% agarose gel and The DNA marker used was DNA Marker (100–2000 bp; B500350 Sangon Biotech (Shanghai) Co., Ltd.), and those with clear bands between 290 and 310 bp were combined for sequencing. The PCR products were then mixed and sequenced on an Illumina HiSeq 2500 platform with the same sequencing information as reported previously^40^.

The sequences were assigned to each of samples based on their unique barcode. All the clean reads were trimmed to a minimum length of 200 and had a Phred score of at least 20 by using the split_libraries_fastq.py script (QIIME 1.9.0)^41^. The reads were clustered into OTUs using the UPARSE strategy^42^ by dereplication with USEARCH 10. All the reads were mapped to their representative sequences using the usearch_global method (USEARCH 10). The OTU table was converted to BIOM format 1.3.1 using BIOM convert for downstream analysis in QIIME 1.9.0. The Greengenes database (V.13.5) was used for taxonomic annotations with the representative sequences. Summary information of the represented taxonomic groups was generated by the use of the summarize_taxa.py script.

### Root exudate collection and GC-TOF-MS detection

The seeds of both cultivars were surface sterilized as mentioned above and then placed in tissue culture bottles (400 mL) containing 100 mL of MS agar media^43^ in a growth chamber (28/26 °C day/night temperature cycle, 70% relative humidity, and 180 μmol light m^−2^ s^−1^) for seven days. Afterward, the sterile seedlings were carefully transferred to conical bottles (one seedling per bottle) containing sterile water and allowed to grow for another seven days. The sterility of the seedlings was tested by plating 100 μL of water from each conical bottle onto an LB^44^ plate and incubating at 30 °C for 3 days. The contaminated seedlings were discarded. Each cultivar was grown as 3 replicates, with 1 replicate including 3 seedlings, that is, 2 cucumber cultivars × 9 individual seedlings, resulting in 18 samples. All the bottles were fully randomized during the experiment. For cucumber growth, we transferred the sterile cucumber seedlings into sterile Hoagland media under gentle shaking (50 rpm) for 2 h each day on a shaker. To collect root exudates, the seedlings were placed in sterile water for three days, and the container was gently shaken as described above. Finally, nine samples of exudates for each cultivar were obtained, stored immediately at −80 °C, and then dried with a lyophilizer (LGJ- 18 S Beijing Songyuanhuaxing Technology Develop Co., Ltd., China).

For extraction, the lyophilized root exudates (with a dose equal to the amount collected from one cucumber seedling) were put into 2 mL EP tubes and then extracted with 1 mL of extraction liquid (*V*_methanol_:*V*_water_ = 3:1), after which 10 μL of adonitol (0.5 mg/mL stock in water) was added as an internal standard; the contents was then mixed for 30 s by vortexing. The mixtures were homogenized in a ball mill for 4 min at 45 Hz, ultrasound treated for 5 min (while incubating in ice water), and centrifuged for 15 min at 13,000 rpm and 4 °C, after which the supernatant (0.75 mL) was transferred to a new 2 mL GC/MS glass vial. After completely drying in a vacuum concentrator without heating, 40 μL of methoxy amination hydrochloride (20 mg/mL in pyridine) was added; the sample was then incubated for 30 min at 80 °C, after which 50 μL of the BSTFA (bis(trimethylsilyl) trifluoroacetamide) reagent (1% TMCS (trimethylchlorosilane), v/v) was added, followed by incubation for 1.5 h at 70 °C. The GC-TOF-MS analysis and raw peak analysis were performed as reported by Li et al.^45^.

### Impacts of four small-molecule organic acids present in the root exudates on the soil microbiome

A soil application experiment was conducted to test the effects of four select small-molecule organic acids (SMOAs; pyruvic acid, citric acid, fumaric acid, and succinic acid) on the soil microbiome. These four compounds were abundant in the FSC root exudate samples. The soil used in the experiment was the same soil as that mentioned above. Organic acid solutions in water were prepared such that they contained each of the selected compounds in equal amounts (2.5 mM citric acid, 2.5 mM pyruvic acid, 2.5 mM succinic acid, and 2.5 mM fumaric acid) and at a final total concentration of 10 mM. Before adding the compound mixture, 15 g of soil was placed into each well of six-well plates. The plates were then preincubated in a growth chamber at 30 °C for 1 week to allow the soil microbiome to acclimate for distinguishing seedling rhizospheres from the bulk soil. Each well then received 1.5 ml of compound mixture solution twice a week for 8½ weeks (17 total applications) in a growth chamber at 30 °C^40^. Sterile ultrapure water was added to each well as a control. Each treatment consisted of 18 wells in three plates. All the plates were randomly arranged during the incubation period. Finally, soils in the 18 wells of each treatment were collected, and three wells were pooled together into one sample. In total, 12 samples for the two treatments (2 treatments × 6 soil samples) were obtained and stored at −80 °C.

For taxonomical profiling of the bacterial communities, 12 samples targeting the V3–V4 region of the 16S rRNA gene were analyzed. The amplification was conducted with the primers 341F/806R (F: CCTAYGGGRBGCASCAG; R: GGACTACHVGGGTWTCTAAT), with an amplicon size of 465 bp. The PCR amplification and 16 S rRNA sequencing were the same as those described above.

Before sequence processing, target sequences were extracted from the raw sequences based on matches to the 515F/806R primers, assuring the same region of the 16 S rRNA gene as that of the rhizosphere samples. Afterward, sequence processing was performed in the same manner as that described above.

### Isolation, identification, and in vitro anti-Foc assays

Strains were isolated from the rhizosphere soil of Foc-susceptible and Foc-resistant cucumber varieties by the dilution plate technique. A 0.5-g aliquot of roots with tightly adhering soil was placed into a 2-mL centrifuge tube containing 1 mL of sterile water and then vortexed at maximum speed for 15 min. Then, 100 μL of soil suspension was used for dilution. Specifically, 100 μL of the soil suspension was pipetted into a dilution tube containing 0.9 mL of sterile water. The tube was subsequently vortexed for ~10 s. After vertexing, 0.1 mL of this solution was removed and placed into a second dilution tube containing 0.9 mL of sterile water. TSA agar was used for all plate media.

Strains were also isolated from SMOA-conditioned and control soils by the dilution plate technique. The soil (0.5 g) was mixed with 5 mL of autoclaved water and placed on an orbital shaker for 30 min, after which 100 μL of soil suspension was used for dilution as described above.

We selected plates with fewer than 100 single colonies, and there was a total of three plates used for each treatment. Then, single colonies were selected and purified twice. In total, 238 single colonies from the FSC rhizosphere and 238 from the FRC rhizosphere were isolated. Each of 189 single colonies was isolated from the SMOA-conditioned soil and that of the control treatment.

DNA extraction, amplification, and sequencing were performed as described by Zhang et al.^46^. The sequences were quality filtered and demultiplexed according to their barcode, and the taxonomy of the sequences was classified using the Greengenes database (V.13.5) as described above.

The antagonistic activity of the isolates against *F. oxysporum* was evaluated with the method reported by Bordoloi et al.^47^. Briefly, a 4-mm agar plug of *F. oxysporum* was placed in the middle of a PDA plate, and the strain was inoculated between the edge of the plate and the plug. The zone of inhibition was measured after incubation at 28 °C for 5 days.

### Statistical methods

For downstream analyses after sequence processing, to describe the rhizosphere community structure, a minimum number of sequences was extracted randomly for each sample to calculate the Shannon index estimated via QIIME by the alpha_diversity.py script. A nonparametric *t*-test was used to determine if the Shannon indices differed between the FRC and FSC. Before calculation of the beta diversity, the cumulative-sum scaling (CSS) method^48^ was used to standardize the OTU profiles by the normalize_table.py script, and Bray-Curtis similarity matrices were prepared using the beta_diversity.py script. Adonis was used to determine whether the beta diversity differed between the two cultivars. Principal coordinate analysis (PCoA) plots were generated from Bray–Curtis similarity matrices created using the R package *ggplot2*. To determine the percent change in taxa, we used *t*-tests for all family- or genus-level taxa with relative abundances >0.001 to measure the significant difference in these abundances between the two cultivars, with the *P*-values corrected according to the Benjamini–Hochberg method. For functional predictions, the UCLUST method was used to select closed-reference operational taxonomic units^49^ at 97% sequence similarity using the Greengenes database (V. 13.5). Functional inferences according to the Kyoto Encyclopedia of Genes and Genomes (KEGG) pathway database were made using PICRUSt^50^. Downstream FishTaco analyses were performed according to the methods of David et al.^51^. Briefly, the top 99 phylotypes with the maximum relative abundance across our OTU table from PICRUSt and normalized with MUSICC were selected. Then, a precomputed OTU-KO table from the PICRUSt analysis, output from MUSICC, and OTU relative abundance table was prepared for input into the FishTaco frame. Multitaxon mode was selected for each pairwise comparison between two cultivar samples. Finally, the R package *ggplot* was used to visualize the function-contribution distribution.

For root exudate analyses, principal component analysis (PCA)^52^ was used to visualize the root exudate structure of the two cultivars using the R package *vegan*. The *p*-values were corrected by the Benjamini–Hochberg FDR procedure for multiple comparisons^53^. To identify the exudate compounds with the greatest contribution to the classification, a random forest approach was applied, and the default parameters in the R implementation of the algorithm (R package *RandomForest*, ntree = 1000) were used. Enrichment analysis of the metabolic pathways was performed and plotted using the online platform MetaboAnalyst^54^ (http://www.metaboanalyst.ca/faces/home.xhtml). All the plots were created using the R package *ggplot2*.

## Supplementary information

Supplementary materials

## Data Availability

The raw sequence data reported in this paper have been deposited in the Genome Sequence Archive of the BIG Data Center, Chinese Academy of Sciences, under accession code CRA002328.
