# Supplementary material for: Enrichment of beneficial cucumber rhizosphere microbes mediated by organic acid secretion
Source: Hortic Res. 2020 Oct 1;7:154. doi: 10.1038/s41438-020-00380-3 (PMC7527982; doi:10.1038/s41438-020-00380-3)
Supplement: Supplementary file 1 — Supplementary materials [file 41438_2020_380_MOESM1_ESM.docx]

## Supplementary Data

**Title:** Enrichment of cucumber rhizosphere beneficial microbes mediated by organic acids secretion

**Authors:**

Supplementary Materials and Method

**Quantification of transcription of plant resistance gene.**

RNA of cucumbers of two cultivars was extracted using RNeasy plant mini kit (Qiagen). Reverse transcription PCR was performed using a PrimeScript RT reagent kit with gDNA eraser (Takara). Real time qPCR was performed using an ABI 7500 Cycler (Applied Biosystems). The 20-μl reaction solution consisted of 10 μl of SYBR Premix EX Taq (2×; TaKaRa), 0.4 μl of each primer (10 μM), 0.4 μl of ROX reference dye II (50×), 2 μl of template DNA, and 6.8 μl of double distilled H2O. The thermal cycling conditions were set to the following: 30 s at 95°C for initial denaturation, followed by 40 cycles of 5 s at 95°C and 34 s at 60°C. The target genes in cucumber plants that encode AOS (Csa2M360780.1; F.primer: CCCGAAACTCAATCACCGTC; R.primer: GAAGGAATCGTAGCGGAGGA), PR2 (Csa1M660200.1; F.primer: TCTTCACAGCTCCGTCAACT; R.primer: TTTCCTCCACCGACCTTCTC), PR3 (Csa6M507520.1; F.primer: GAGACTACAGGAGGATGGCC; R.primer: TTGTTGACCAGAAGCACACG), and PR4 (Csa6M507520.1; F.primer: GAGACTACAGGAGGATGGCC; R.primer: TTGTTGACCAGAAGCACACG), were quantified using an ACT1 gene (F.primer: GTGGTGGTGAATGAGTAGCC; R.primer: TTGGATTCTGGTGATGGTGTC) as an internal reference (Liu et al. 2017); the primers are shown in supplementary table S11.

Supplementary Table 1. Relative abundance (%) of specific family enriched in the rhizosphere soil of FSC and the entire family present here were significant different (p<0.05, t-test) between two cultivars.

| Tax | ra_FSC | ra_FRC | sd_FSC | sd_FRC | log2_FC | Pvalue | fdr | level |
| --- | --- | --- | --- | --- | --- | --- | --- | --- |
| Comamonadaceae | 0.199 | 0.095 | 0.044 | 0.038 | 1.061 | 0.001 | 0.001 | enriched |
| Xanthomonadaceae | 0.102 | 0.048 | 0.014 | 0.016 | 1.086 | 0.000 | 0.000 | enriched |
| Caulobacteraceae | 0.075 | 0.034 | 0.016 | 0.015 | 1.096 | 0.001 | 0.001 | enriched |
| Rhizobiaceae | 0.031 | 0.020 | 0.007 | 0.009 | 0.654 | 0.029 | 0.029 | enriched |
| Cytophagaceae | 0.022 | 0.015 | 0.005 | 0.003 | 0.537 | 0.020 | 0.020 | enriched |
| Rhodocyclaceae | 0.005 | 0.003 | 0.001 | 0.001 | 0.567 | 0.001 | 0.001 | enriched |
| Erythrobacteraceae | 0.005 | 0.003 | 0.000 | 0.001 | 0.385 | 0.001 | 0.001 | enriched |
| Haliangiaceae | 0.002 | 0.001 | 0.001 | 0.000 | 0.645 | 0.018 | 0.018 | enriched |
| Nannocystaceae | 0.002 | 0.001 | 0.001 | 0.000497 | 0.661 | 0.043 | 0.043 | enriched |

ra: relative abundance

Supplementary Table 2. Relative abundance (%) of specific genus enriched in the rhizosphere soil of FSC and the entire genus present here were significant different (p<0.05, t-test) between two cultivars.

| Tax | RA_FSC | RA_FRC | log2_FC | Pvalue | fdr | level | SD_FSC | SD_FRC |
| --- | --- | --- | --- | --- | --- | --- | --- | --- |
| X.Pseudoxanthomonas | 0.031 | 0.009 | 1.620 | 0.002 | 0.002 | enriched | 0.010 | 0.007 |
| X.Lysobacter | 0.011 | 0.002 | 1.819 | 0.000 | 0.000 | enriched | 0.003 | 0.001 |
| X.Luteimonas | 0.010 | 0.004 | 0.974 | 0.008 | 0.008 | enriched | 0.003 | 0.001 |
| X.Thermomonas | 0.009 | 0.006 | 0.629 | 0.029 | 0.029 | enriched | 0.003 | 0.002 |
| S.Steroidobacter | 0.002 | 0.001 | 0.469 | 0.044 | 0.044 | enriched | 0.001 | 0.000 |
| X.Stenotrophomonas | 0.002 | 0.000 | 1.215 | 0.036 | 0.036 | enriched | 0.001 | 0.000 |
| P.Pseudomonas | 0.001 | 0.001 | 0.549 | 0.003 | 0.003 | enriched | 0.000 | 0.000 |
| C.Methylibium | 0.006 | 0.004 | 0.623 | 0.001 | 0.001 | enriched | 0.001 | 0.001 |
| C.Hydrogenophaga | 0.004 | 0.001 | 0.939 | 0.004 | 0.004 | enriched | 0.001 | 0.001 |
| C.Rubrivivax | 0.003 | 0.002 | 0.401 | 0.001 | 0.001 | enriched | 0.000 | 0.000 |
| C.Phenylobacterium | 0.034 | 0.018 | 0.870 | 0.001 | 0.001 | enriched | 0.006 | 0.005 |
| C.Caulobacter | 0.016 | 0.005 | 1.460 | 0.009 | 0.009 | enriched | 0.007 | 0.003 |
| C.Asticcacaulis | 0.014 | 0.005 | 1.299 | 0.004 | 0.004 | enriched | 0.004 | 0.004 |
| R.Agrobacterium | 0.005 | 0.002 | 0.950 | 0.001 | 0.001 | enriched | 0.001 | 0.001 |
| S.Sphingopyxis | 0.001 | 0.001 | 0.616 | 0.004 | 0.004 | enriched | 0.000 | 0.000 |
| C.Niastella | 0.033 | 0.016 | 0.979 | 0.021 | 0.021 | enriched | 0.008 | 0.012 |
| N.Nannocystis | 0.001 | 0.001 | 0.702 | 0.039 | 0.039 | enriched | 0.001 | 0.001 |

Note: ra: relative abundance

Supplementary Table 3. The number of sixteen different isolates each isolates from the treatment of FSC and FRC, which belonging to *Bacillaceae*, *Pseudomonadaceae*, *Comamonadaceae*, *Xanthomonadgaceae*, *Enterobacteriaceae*, *Oxalobacteraceae* and *Weeksellaceae*.

| ID^a^ | Phylum | Family | Genus | FSC^b^ | FRC^c^ |
| --- | --- | --- | --- | --- | --- |
| G41 | Firmicutes | Bacillaceae | Bacillus | 12 | 15 |
| T24 | Proteobacteria | Xanthomonadaceae | Lysobacter | 6 | 2 |
| G47 | Proteobacteria | Xanthomonadaceae | Stenotrophomonas | 30 | 26 |
| W16 | Proteobacteria | Pseudomonadaceae | Pseudomonas | 13 | 16 |
| F19 | Proteobacteria | Oxalobacteraceae | Ralstonia | 16 | 18 |
| CN11 | Firmicutes | Bacillaceae | Bacillus | 27 | 18 |
| FM2 | Proteobacteria | Comamonadaceae | — | 18 | 12 |
| G11 | Proteobacteria | Comamonadaceae | — | 22 | 12 |
| F7 | Proteobacteria | Oxalobacteraceae | — | 7 | 18 |
| FG25 | Firmicutes | Bacillaceae | Bacillus | 16 | 10 |
| F32 | Firmicutes | Bacillaceae | Bacillus | 36 | 45 |
| M8 | Proteobacteria | Pseudomonadaceae | Pseudomonas | 11 | 9 |
| G8 | Proteobacteria | Enterobacteriaceae | — | 2 | 0 |
| W46 | Proteobacteria | Xanthomonadaceae | Stenotrophomonas | 4 | 7 |
| FT14 | Bacteroidetes | Weeksellaceae | Chryseobacterium | 3 | 11 |
| FT11 | Proteobacteria | Oxalobacteraceae | Ralstonia | 15 | 19 |

a: Name the single colonies of the same sequence

b and c: Count the number of single colonies of the same sequence from total of 238 single colonies from the FSC rhizosphere or FRC rhizosphere

Supplementary Table 4. The top 10 (top 4% of total 236 compounds) compounds sorted by loading variables importance of loading matrix of the PC1 axis (81.9% of variance explained) from Principal components analysis (PCA).

| ID | PC1 | classification |
| --- | --- | --- |
| (2R,3S)-2-hydroxy-3-isopropylbutanedioic acid | 0.08 | others |
| Fructose-6-phosphate | 0.08 | others |
| N-Acetyl-beta-alanine 1 | 0.08 | amino acids and amides |
| Pyruvic acid | 0.08 | short chain carbon organic acids |
| Urocanic acid 2 | 0.08 | others |
| Oxamide | 0.08 | others |
| Ascorbate | 0.08 | esters |
| Lactic acid | 0.08 | short chain carbon organic acids |
| N-Carbamylglutamate 4 | 0.08 | amino acids and amides |
| Dihydroxyacetone | 0.08 | sugars |

PC1: loading of the first axis ordered by important by PCA;

Supplementary Table 5. The top 35 (top 15% of total 236 compounds) compounds sorted by loading variables importance from random forests classification.

| Compounds | MeanDecreaseAccuracy ^a^ |
| --- | --- |
| Succinic acid | 2.41 |
| Ribonic acid, gamma-lactone | 2.21 |
| 2-amino-3-(4-hydroxyphenyl)propanoic acid | 2.2 |
| Methyl Palmitoleate | 2.2 |
| Glucosaminic acid | 1.98 |
| Linoleic acid methyl ester | 1.97 |
| Allantoic acid | 1.97 |
| 3-hydroxy-L-proline | 1.96 |
| Methyl-beta-D-galactopyranoside | 1.96 |
| D-Glyceric acid | 1.96 |
| Melibiose | 1.94 |
| 2,3-Dihydroxypyridine | 1.73 |
| Isoxanthopterin | 1.73 |
| Trehalose | 1.73 |
| Ethanolamine | 1.73 |
| Prostaglandin A2 | 1.73 |
| Conduritol b epoxide | 1.71 |
| Isoleucine | 1.71 |
| Sulfuric acid | 1.71 |
| Threonic acid | 1.71 |
| 2-hydroxypyridine | 1.71 |
| 4-hydroxybutyrate | 1.71 |
| Hydroquinone | 1.71 |
| Quinic acid | 1.71 |
| DL-Anabasine | 1.7 |
| Octadecanol | 1.7 |
| Capric Acid | 1.7 |
| Galactinol | 1.7 |
| Indole-3-acetamide | 1.7 |
| Threonine | 1.7 |
| Fumaric acid | 1.7 |
| Cytidine-monophosphate degr prod | 1.67 |
| Shikimic acid | 1.67 |
| Citric acid | *1.42* |

^a^ MeanDecreaseAccuracy: increase in mean squared error (%)

Supplementary Table 6. 34 compounds were collected and that is representing the main difference of root exudates between two cultivars using PCA loading matrix and random forests classification approach.

| ID | source | rs_FSC | rs_FRC | tax |
| --- | --- | --- | --- | --- |
| Dihydroxyacetone | PC1 | 0.000 | 0.932 | sugars |
| Ribonic acid, gamma-lactone | RF | 0.036 | 0.034 | sugar acids |
| Citric acid | RF | 0.053 | 0.030 | short chain carbon organic acids |
| Pyruvic acid | PC1 | 0.049 | 0.000 | short chain carbon organic acids |
| Fumaric acid | RF | 0.036 | 0.037 | short chain carbon organic acids |
| D-Glyceric acid | RF | 0.286 | 0.118 | short chain carbon organic acids |
| Succinic acid | RF | 1.735 | 1.534 | short chain carbon organic acids |
| Lactic acid | PC1 | 11.089 | 31.508 | short chain carbon organic acids |
| Shikimic acid | RF | 0.252 | 1.195 | others |
| 2-amino-3-(4-hydroxyphenyl) propanoic acid | RF | 0.034 | 0.030 | others |
| Allantoic acid | RF | 0.000 | 0.027 | others |
| Oxamide | PC1 | 0.000 | 0.036 | others |
| (2R,3S)-2-hydroxy-3-isopropylbutanedioic acid | PC1 | 0.000 | 0.034 | others |
| urocanic acid | PC1 | 0.000 | 0.034 | others |
| Methyl-beta-D-galactopyranoside | RF | 0.000 | 0.000 | others |
| Glucosaminic acid | RF | 0.000 | 0.003 | others |
| Isoxanthopterin | RF | 0.000 | 0.000 | others |
| Hydroquinone | RF | 0.017 | 0.018 | others |
| Indole-3-acetamide | RF | 0.014 | 0.010 | others |
| DL-Anabasine | RF | 0.000 | 0.000 | others |
| Conduritol b epoxide | RF | 0.052 | 0.103 | others |
| Prostaglandin A2 | RF | 0.000 | 0.000 | others |
| Fructose-6-phosphate | PC1 | 0.000 | 0.005 | others |
| Cytidine-monophosphate degr prod | RF | 0.000 | 0.000 | others |
| 2-hydroxypyridine | RF | 0.053 | 0.033 | others |
| Capric Acid | RF | 0.000 | 0.000 | long chain carbon organic acids |
| Ascorbate | PC1 | 0.000 | 0.048 | esters |
| 4-hydroxybutyrate | RF | 0.308 | 1.758 | esters |
| N-Acetyl-beta-alanine | PC1 | 0.000 | 0.041 | amino acids and amides |
| Isoleucine | RF | 0.000 | 0.010 | amino acids and amides |
| Threonine 1 | RF | 0.000 | 0.027 | amino acids and amides |
| N-Carbamylglutamate | PC1 | 0.000 | 0.006 | amino acids and amides |
| Galactinol | RF | 0.000 | 0.000 | alcohols |
| Octadecanol | RF | 0.000 | 0.000 | alcohols |

Note: rs: relative abundance

Supplementary Table 7. Mentel's test to root exudates and rhizosphere communities of two cultivars were significantly associated with rhizosphere microorganisms

| method | pearson |
| --- | --- |
| Mantel statistic r | 0.5966 |
| Significance (p) | 0.003 |

Supplementary Table 8. The difference in metabolic pathways caused by the root exudates of the two cucumbers.

|  | Total Cmpd | Hits | Raw p | -LOG(p) | Holm adjust | Impact |
| --- | --- | --- | --- | --- | --- | --- |
| Ascorbate and aldarate metabolism | 14 | 1 | 0.000 | 25.928 | 0.000 | 1.000 |
| beta-Alanine metabolism | 12 | 3 | 0.000 | 8.317 | 0.006 | 0.436 |
| Glyoxylate and dicarboxylate metabolism | 17 | 3 | 0.000 | 10.463 | 0.001 | 0.239 |
| Glycine, serine and threonine metabolism | 29 | 2 | 0.000 | 10.820 | 0.001 | 0.218 |
| Citrate cycle (TCA cycle) | 20 | 4 | 0.000 | 11.703 | 0.000 | 0.175 |
| Pantothenate and CoA biosynthesis | 16 | 4 | 0.000 | 9.854 | 0.001 | 0.162 |
| Pyruvate metabolism | 20 | 2 | 0.000 | 12.994 | 0.000 | 0.149 |
| Glycolysis or Gluconeogenesis | 25 | 3 | 0.000 | 12.611 | 0.000 | 0.121 |
| Glycerolipid metabolism | 14 | 2 | 0.000 | 10.901 | 0.001 | 0.094 |
| Galactose metabolism | 26 | 4 | 0.000 | 8.840 | 0.004 | 0.093 |
| Phenylalanine, tyrosine and tryptophan biosynthesis | 22 | 1 | 0.000 | 10.248 | 0.001 | 0.085 |
| Pyrimidine metabolism | 39 | 5 | 0.000 | 7.860 | 0.008 | 0.057 |
| Carbon fixation in photosynthetic organisms | 21 | 2 | 0.000 | 14.351 | 0.000 | 0.042 |
| Valine, leucine and isoleucine biosynthesis | 26 | 3 | 0.000 | 18.813 | 0.000 | 0.036 |
| Cysteine and methionine metabolism | 35 | 2 | 0.001 | 7.191 | 0.016 | 0.014 |
| Alanine, aspartate and glutamate metabolism | 21 | 3 | 0.000 | 8.510 | 0.005 | 0.007 |

Supplementary Table 9. Ten compounds selected by PCA, random forest classification and pathway enrichment analyses that were significantly (t-test, p<0.05) differential in relative abundance between two root exudate profiles.

| ID | KEGG | meanCSF | meanCRF | from | log2_FC | tax |
| --- | --- | --- | --- | --- | --- | --- |
| ascorbate | C00072 | 0 | 116588.9 | PC1 | -5.62477 | esters |
| lactic acid | C00186 | 9554238 | 76060491 | PC1 | -1.50647 | short chain carbon organic acids |
| Pyruvic acid | C00022 | 42528.03 | 0 | PC1 | 5.656452 | short chain carbon organic acids |
| citric acid | C00158 | 82789.92 | 0 | RF | 6.609756 | short chain carbon organic acids |
| cytidine-monophosphate degr prod | C00055 | 103067.5 | 77667.76 | RF | 1.867903 | others |
| D-Glyceric acid | C00258 | 469793.9 | 365274.8 | RF | 1.841035 | short chain carbon organic acids |
| fumaric acid | C00122 | 70560.43 | 31275.05 | RF | 2.57027 | amino acids and amides |
| Isoleucine | C00407 | 0 | 58615.15 | RF | -4.66048 | amino acids and amides |
| shikimic acid | C00493 | 0 | 799925.4 | RF | -8.37488 | others |
| succinic acid | C00042 | 1672082 | 1330090 | RF | 1.808923 | short chain carbon organic acids |

Supplementary Table 10. T-text of the rhizosphere microbiome between the four small molecule organic acids (SMOAs) and control revealed a distinct differential abundance of specific family group.

| Taxa | ra_SMOAs | sa_SFAS | ra_control | sd_control | log2_FC | Pvalue | fdr |
| --- | --- | --- | --- | --- | --- | --- | --- |
| f__Rhodobacteraceae | 0.001 | 0.000 | 0.000 | 0.000 | 0.393 | 0.007 | 0.007 |
| f__Burkholderiaceae | 0.004 | 0.002 | 0.000 | 0.000 | 1.608 | 0.009 | 0.009 |
| f__Comamonadaceae | 0.036 | 0.007 | 0.007 | 0.001 | 2.224 | 0.000 | 0.000 |
| f__Oxalobacteraceae | 0.010 | 0.003 | 0.001 | 0.000 | 2.465 | 0.001 | 0.001 |
| f__Sinobacteraceae | 0.005 | 0.001 | 0.010 | 0.002 | -0.785 | 0.000 | 0.000 |
| f__Xanthomonadaceae | 0.009 | 0.001 | 0.012 | 0.002 | -0.407 | 0.010 | 0.010 |

ra: relative abundance

Supplementary Table 11. The number of fourteen different isolates from the SMOAs conditioned and control soils, which belong to *Bacillaceae*, *Pseudomonadaceae*, *Comamonadaceae*, *Xanthomonadgaceae*, *Sphingomonadaceae*, *Burkholderiaceae*, *Alcaligenaceae*, *Oxalobacteraceae*, and *Rhizobiaceae*.

| ID^a^ | ID2^b^ | Phylum | Family | Genus | SMOAs^c^ | CK^d^ |
| --- | --- | --- | --- | --- | --- | --- |
| Y1 | G41 | Firmicutes | Bacillaceae | Bacillus | 18 | 16 |
| Y2 | F19 | Proteobacteria | Oxalobacteraceae | Ralstonia | 9 | 11 |
| G43 | G11 | Proteobacteria | Comamonadaceae |  | 30 | 12 |
| G86 | FG25 | Firmicutes | Bacillaceae | Bacillus | 33 | 29 |
| G53 | F32 | Firmicutes | Bacillaceae | Bacillus | 22 | 26 |
| Y45 | W46 | Proteobacteria | Xanthomonadaceae | Stenotrophomonas | 4 | 6 |
| P42 | —— | Proteobacteria | Pseudomonadaceae | Pseudomonas | 13 | 19 |
| P73 | —— | Proteobacteria | Pseudomonadaceae | Pseudomonas | 8 | 16 |
| P89 | —— | Proteobacteria | Sphingomonadaceae | Sphingomonas | 9 | 5 |
| BT4 | —— | Proteobacteria | Burkholderiaceae | Burkholderia | 14 | 16 |
| BT12 | —— | Proteobacteria | Burkholderiaceae | Burkholderia | 17 | 20 |
| F43 | —— | Proteobacteria | Alcaligenaceae | Alcaligenes | 3 | 13 |
| W33 | —— | Proteobacteria | Rhizobiaceae | Rhizobium | 9 | 0 |

a: Name the single colonies of the same sequence

b: 16s rRNA gene V5-V7 sequence of the strains from the FSC rhizosphere or FRC rhizosphere were the same as strains from SMOAs or CK treatment. “—” There were no strain which with the same 16s rRNA gene V5-V7 sequence with the first column same row from the FSC rhizosphere or FRC rhizosphere

c and d: Count the number of single colonies of the same sequence from total of 189 single colonies from SMOAs or CK treatment.

Supplementary Table 12. Transcription of genes involved in plant resistance in two cultivars.

|  | FRC (Ct by normalized using ACT1) | | | FSC (Ct by normalized using ACT1) | | | | fold_change (2^-△△Ct1^) |
| --- | --- | --- | --- | --- | --- | --- | --- | --- |
|  | Repeat 1 | Repeat 2 | Repeat 3 | | Repeat 1 | Repeat 2 | Repeat 3 |  |
| AOS | 8.96 | 8.24 | 8.70 | | 8.64 | 9.04 | 9.12 | 0.86 |
| PR2 | 5.54 | 5.69 | 5.16 | | 0.01 | 0.30 | 0.78 | 43.77 |
| PR3 | 5.98 | 5.64 | 5.59 | | 0.42 | 0.32 | 0.37 | 61.22 |
| PR4 | 5.60 | 5.94 | 5.57 | | 0.43 | 0.41 | 0.22 | 62.62 |

AOS, allene oxide synthase (Csa2M360780.1); PR1, PR protein 1 (Csa003482); PR2, PR protein 2 (Csa1M660200.1); PR3, PR protein 3 (Csa6M507520.1); PR4, pathogenesis-related PR protein 4 (Csa6M507520.1). All genes were normalized using ACT1 (actin) as reference.

Supplementary Figure 1*.* Marked up-regulation of protein synthesis and secretion and bacterial chemotaxis in functional of FSC, and deconvolution of significant community-wide functional shifts into individual taxonomic contributions. The right bar plot represents relative contributions driving functional shifts by the taxa of Foc-susceptible samples. The left barplot represents relative contributions reducing functional shifts by the taxa of Foc-susceptible samples.


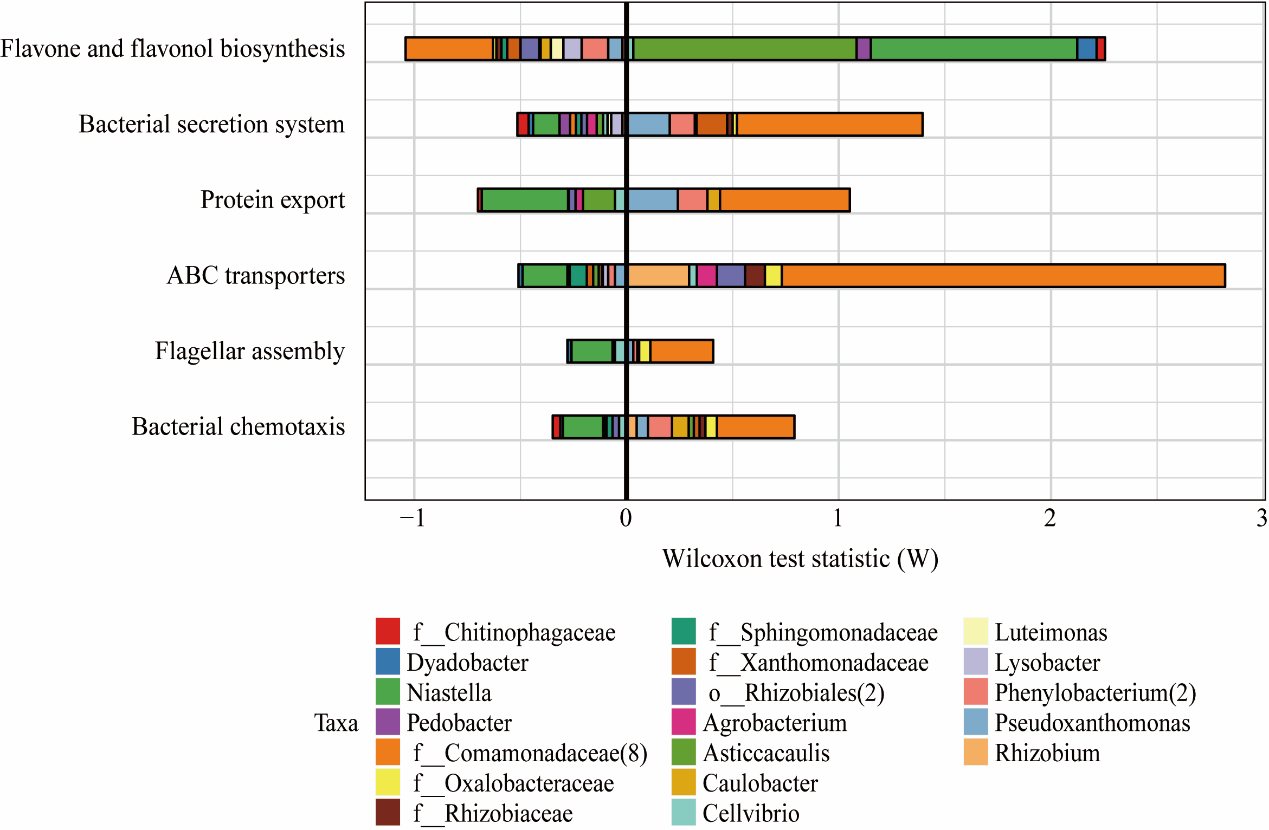


Supplementary Figure 2. Up-regulation of amino acid metabolism in FSC, and deconvolution of significant community-wide functional shifts into individual taxonomic contributions. The right barplot represents relative contributions driving functional shifts by the taxa of Foc-susceptible samples. The left barplot represents relative contributions reducing functional shifts by the taxa of Foc-susceptible samples.


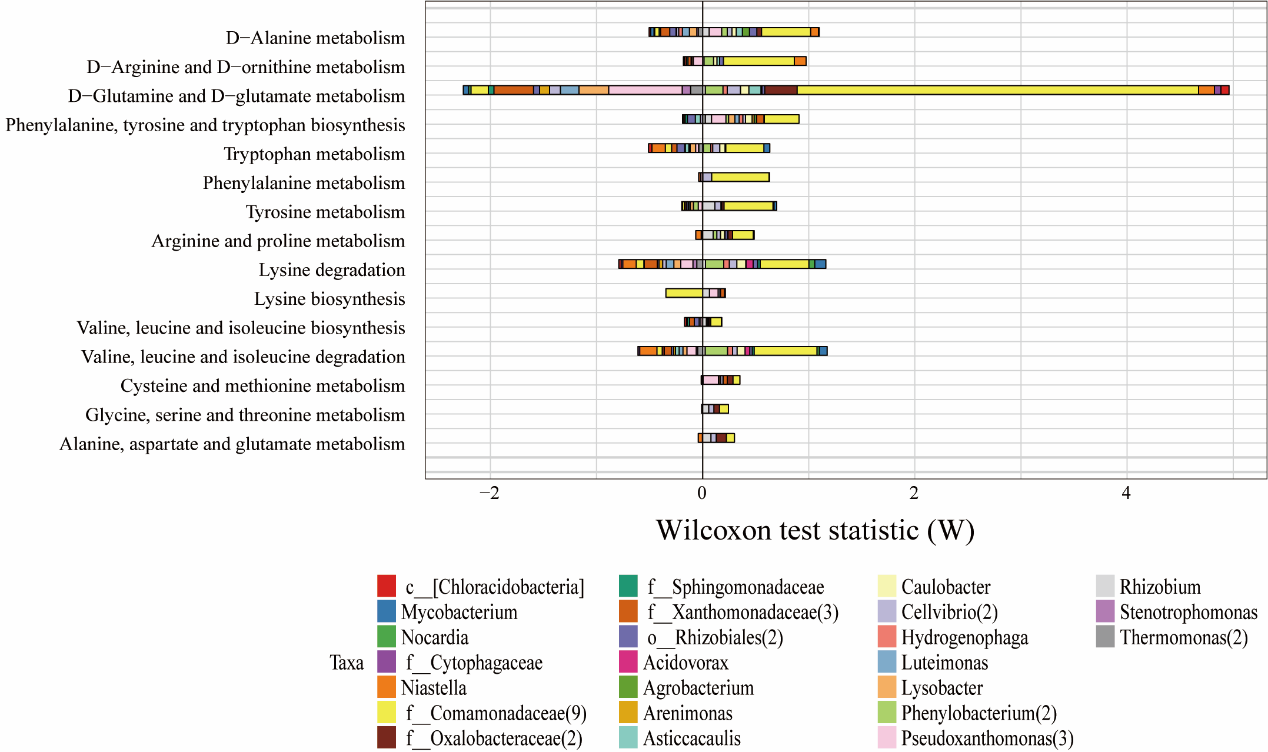


Reference:

Liu, Y., L. Chen, G. Wu, H. Feng, G. Zhang, Q. Shen & R. Zhang (2017) Identification of Root-Secreted Compounds Involved in the Communication Between Cucumber, the Beneficial Bacillus amyloliquefaciens, and the Soil-Borne Pathogen Fusarium oxysporum. *Mol Plant Microbe Interact,* 30**,** 53-62.
